# Supplementary material for: HSP47 levels determine the degree of body adiposity
Source: Nat Commun. 2023 Nov 11;14:7319. doi: 10.1038/s41467-023-43080-x (PMC10640548; doi:10.1038/s41467-023-43080-x)
Supplement: Supplementary file 1 — Supplementary Information [file 41467_2023_43080_MOESM1_ESM.pdf]

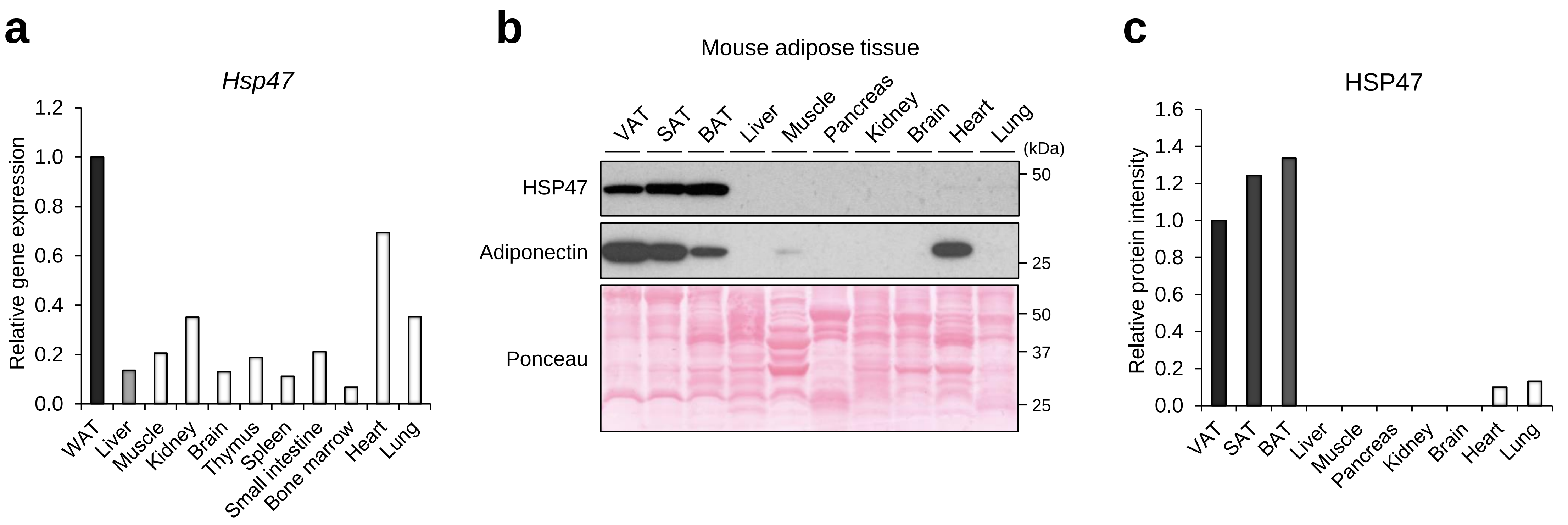

Supplementary Figure 1. HSP47 gene and protein expression in mouse tissues. a, Relative Hsp47 gene expression in white adipose tissue (epididymal), liver, gastrocnemius muscle, kidney, brain, heart, thymus, spleen, small intestine, bone marrow, heart, and lung of C57/BL6 mice (Male; 10-12 weeks old; GDS3142). b and c, Western blot image (b) of HSP47, Adiponectin, and ponceau staining in VAT (epididymal fat; visceral adipose tissue), SAT (inguinal fat; subcutaneous adipose tissue), BAT (interscapular brown fat; brown adipose tissue), liver, gastrocnemius muscle, pancreas, kidney, brain, heart, and lung of C57/BL6J mice; the densitometry (c) of HSP47 protein. Source data are provided as a Source Data file.

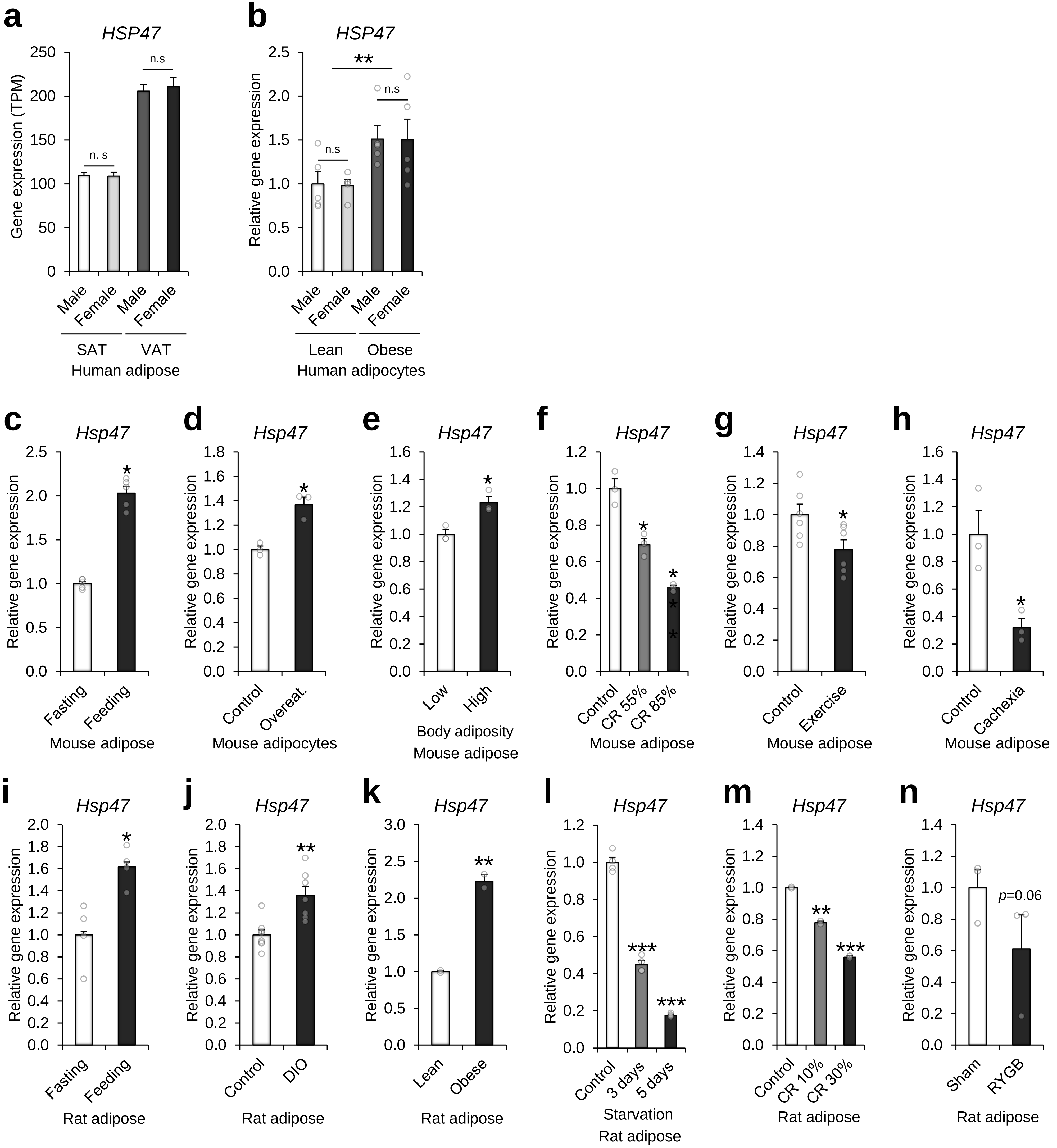

Supplementary Figure 2. The gene expression of HSP47 in human, mouse, and rat adipose tissue. a, Gene expression (TPM) of HSP47 in human subcutaneous (SAT; male n=445, female n=218) and visceral adipose tissue (VAT; male n=371, female 170) from male and female subjects (GTEx). b, Relative gene expression of HSP47 in human lean and obese subcutaneous adipocytes from male and female subjects (GDS3602; n=5 each; lean vs obese, p=0.0029). c, Mouse adipose tissue under fasting and feeding conditions (GDS4918, n=5 each, 24 hours fasting or ad libitum feeding; p=0.00000118). d, Mouse adipocytes after control and overeating diet (GDS5824, n=3 each, normal chow control or high-fat diet for 3 days; p=0.005769). e, Mouse adipose tissue from low- or high-weight gainer group after 3 months of high-fat diet (GDS2319, n=3 each; p=0.0143). f, Mouse adipose tissue from after 10 weeks of control, 55% (control vs 55%, p=0.008657), and 85% (control vs 85%, p=0.000558) calorie restriction diets (GSE60596, n=3 each). g, Mouse adipose tissue housed in control or running wheel cages for 10 weeks (GSE70857, n=6 each; p=0.0361). h, Mouse adipose tissue from control or a pancreatic cancer-induced cachexia model (GDS4899, n=3 each, p=0.0215). i, Rat adipose tissue under fasting or feeding conditions (GDS3135, n=4 each, 24 hours fasting or ad libitum feeding; p=0.0109). j, Rat adipose tissue from lean control and diet-induced obesity (DIO) model (GDS2946, normal chow control n=8 or high-fat/high-sucrose diet for about 6 month; p=0.001731).k, Rat adipose tissue from 4 months old of WNIN/Ob lean and obese rats (GSE58575, n=2 each, p=0.005662). l, Rat adipose tissue after control, 3 days (p=0.000003903), and 5 days (p=0.000000103) of starvation (GSE118978, n=3 each). m, Rat adipose tissue after 1 month of control, 10% (p=0.002346), and 30% (p=0.000357) calorie restriction (GSE176295, n=2 each). n, Rat adipose tissue at 90 days after sham or Roux-en-Y gastric bypass (RYGB) surgery (GDS2956, n=3 each, pair fed). Data represent the mean ± SEM. *$p < 0.05$, **$p < 0.01$, and ***$p < 0.001$; n refers to sample size. Statistical significance was determined by two-tailed unpaired t-test. Source data are provided as a Source Data file.

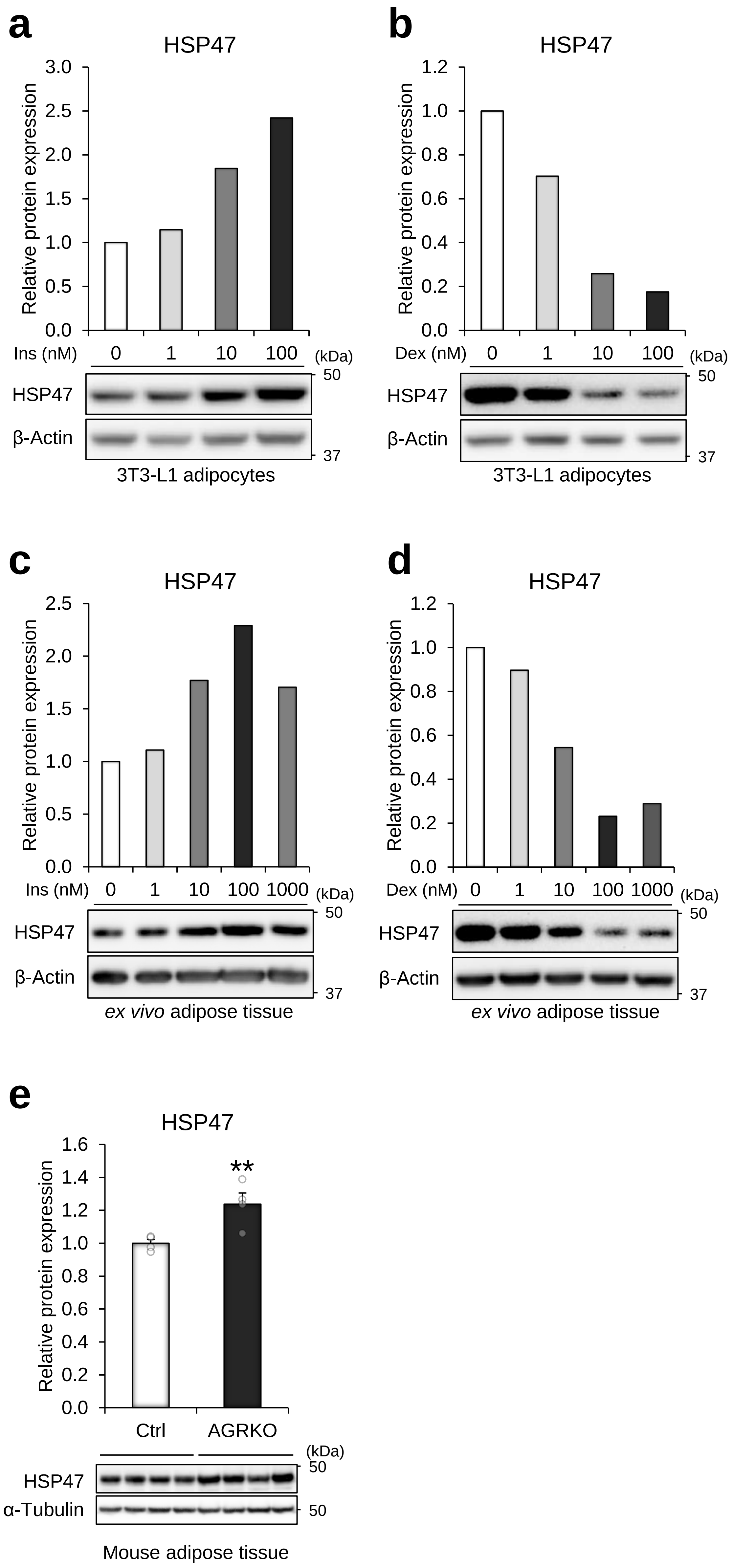

Supplementary Figure 3. HSP47 regulation in adipocytes and adipose tissue. a and b, Relative protein expression and western blot image of HSP47 in 3T3-L1 adipocytes after 48 hours of insulin (a; ins; 0, 1, 10, and 100 nM) or dexamethasone (b; Dex 0, 1, 10, and 100 nM) treatment. c and d, Relative protein expression and western blot image of HSP47 in ex vivo adipose explant after 48 hours of insulin (c; ins; 0, 1, 10, 100, and 1000 nM) or dexamethasone (d; Dex 0, 1, 10, 100, and 1000 nM) treatment. e, Relative protein expression and western blot image of HSP47 protein in mouse adipose tissues of control (Ctrl; flox/flox) or adipose-specific GR knockout (AGRKO; flox/flox and Adipoq-Cre) mice under normal diet condition (n=4). Data represent the mean ± SEM. **$p < 0.01$; n refers to sample size. Statistical significance was determined by two-tailed unpaired t-test. Source data are provided as a Source Data file.

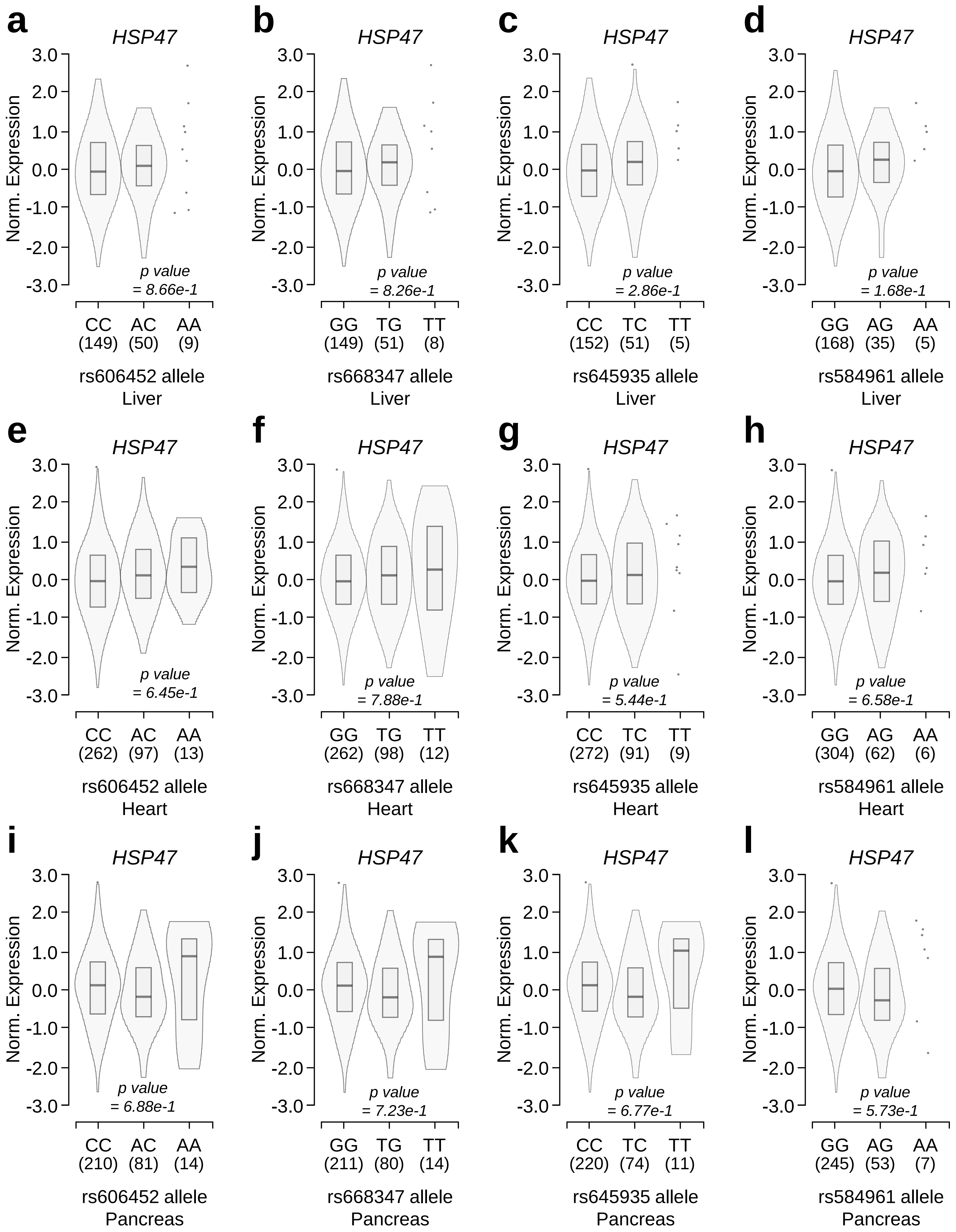

Supplementary Figure 4. eQTL analysis of HSP47 genetic variants in liver, heart and pancreas. a-d, eQTL analyses of genetic variants rs606452 (a; genotype CC n=149; AC n=50; AA n=9), rs668347 (b; genotype GG n=149; TG n=51; TT n=8), rs645935 (c; genotype CC n=152; TC n=51; TT n=5), rs584961 (d; genotype n=168; AG n=35; AA n=5) in human liver tissue. e-h, eQTL analyses of genetic variants rs606452 (e), rs668347 (f), rs645935 (g), and rs584961 (h) in human heart tissue. i-l, eQTL analyses of genetic variants rs606452 (i), rs668347 (j), rs645935 (k), and rs584961 (l) in human pancreas tissue. Box plots are shown as median and 25th and 75th percentiles; points are displayed as outliers if they are above or below 1.5 times the interquartile range. Statistical p-value is from a t-test that compares observed normalized enrichment score (NES) from single-tissue eQTL analysis to a null NES of 0; n refers to sample size.

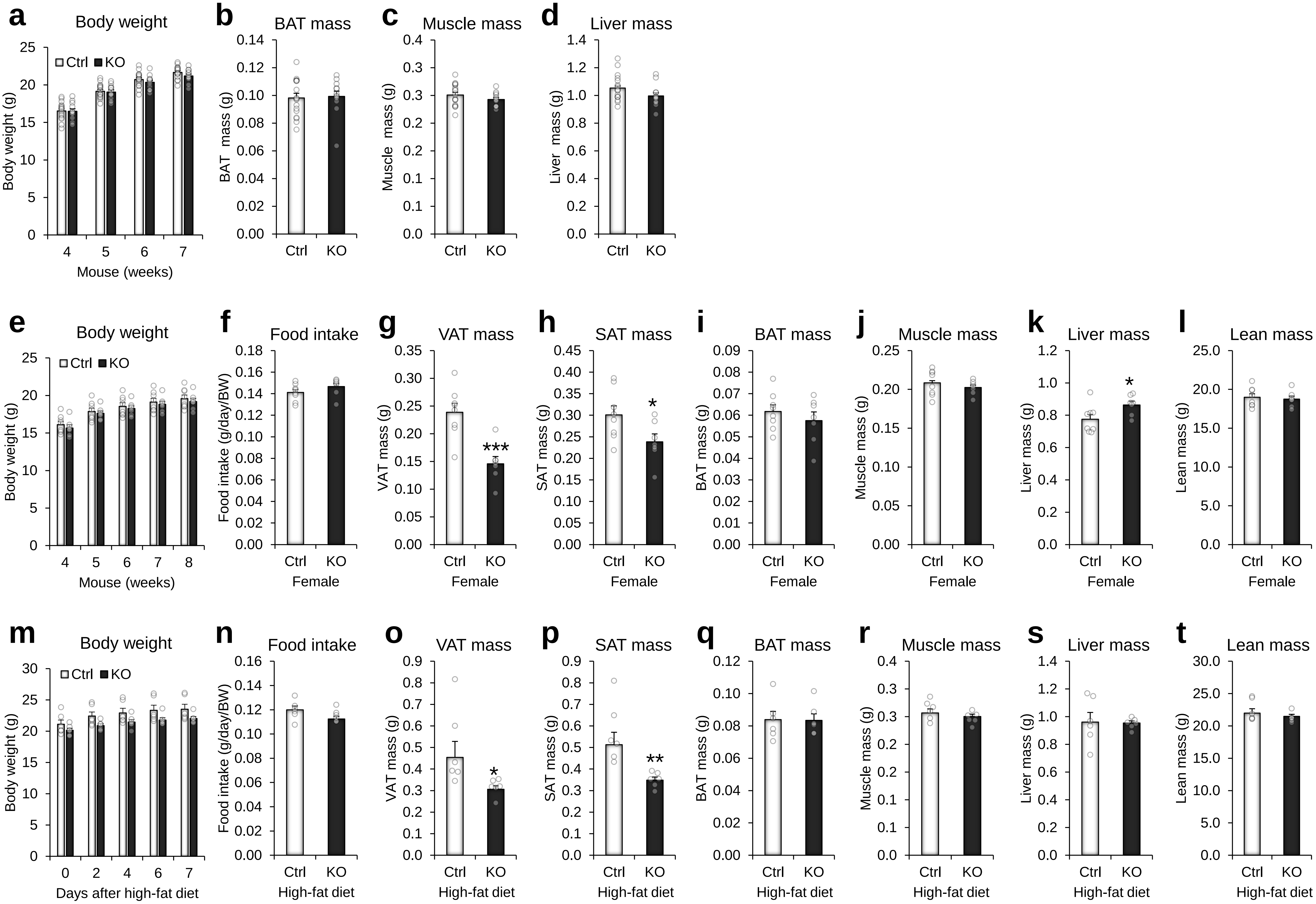

Supplementary Figure 5. lower body adiposity in AdHSP47KO mice. a-d, Body weight (a), brown adipose tissue (b; BAT), skeletal muscle (c), and liver (d) mass of control (flox/flox) and AdHSP47KO (flox/flox; Adipoq-Cre) mice under normal diet (Control n=17; AdHSP47KO n=12). e-l, Body weight (e), food intake (f), visceral adipose tissue (g; VAT; periovarian fat; p=0.00062), subcutaneous adipose tissue (h; SAT; inguinal fat; p=0.044), brown adipose tissue (i; BAT), skeletal muscle (j), liver (k; p=0.0407), lean (l) mass of female control (n=8) and AdHSP47KO mice (n=7). m-t, Body weight (m), food intake (n), visceral adipose tissue (o; VAT; epididymal fat; p=0.0384), subcutaneous adipose tissue (p; SAT; inguinal fat; p=0.0045), brown adipose tissue (q; BAT), skeletal muscle (r), liver (s), lean (t) mass of male control and AdHSP47KO mice under 7 days of high-fat diet (n=7 each). Data represent the mean ± SEM. *p < 0.05, **p < 0.01, and ***p < 0.001; n refers to sample size. Statistical significance was determined by two-tailed unpaired t-test. Source data are provided as a Source Data file.

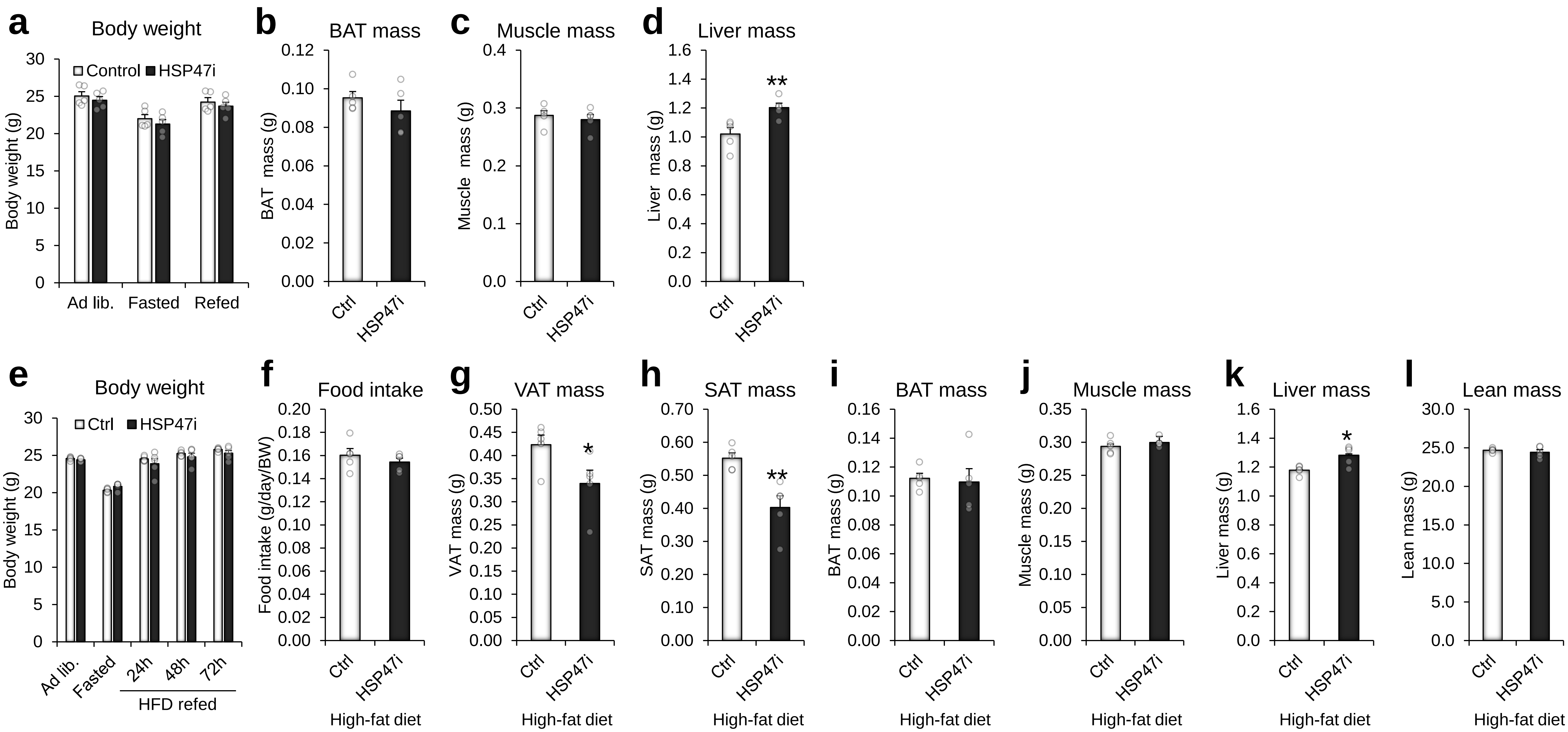

Supplementary Figure 6. lower body adiposity in HSP47i mice. a-d, Body weight (a), brown adipose tissue (b; BAT), skeletal muscle (c), and liver (d; p=0.0098) mass of control and HSP47i mice (n=5 each) under 24 hours of normal diet refeeding condition after 24 hours fasting. e-l, Body weight (e), food intake (f), visceral adipose tissue (g; VAT; periovarian fat; p=0.046), subcutaneous adipose tissue (h; SAT; inguinal fat; p=0.0047), brown adipose tissue (i; BAT), skeletal muscle (j), liver (k; p=0.014), lean (l) mass of control and HSP47i mice (n=5 each) under 3 days of high-fat diet refeeding after 24 hours fasting. Data represent the mean ± SEM. *$p < 0.05$, **$p < 0.01$, and ***$p < 0.001$; n refers to sample size. Statistical significance was determined by two-tailed unpaired t-test. Source data are provided as a Source Data file.

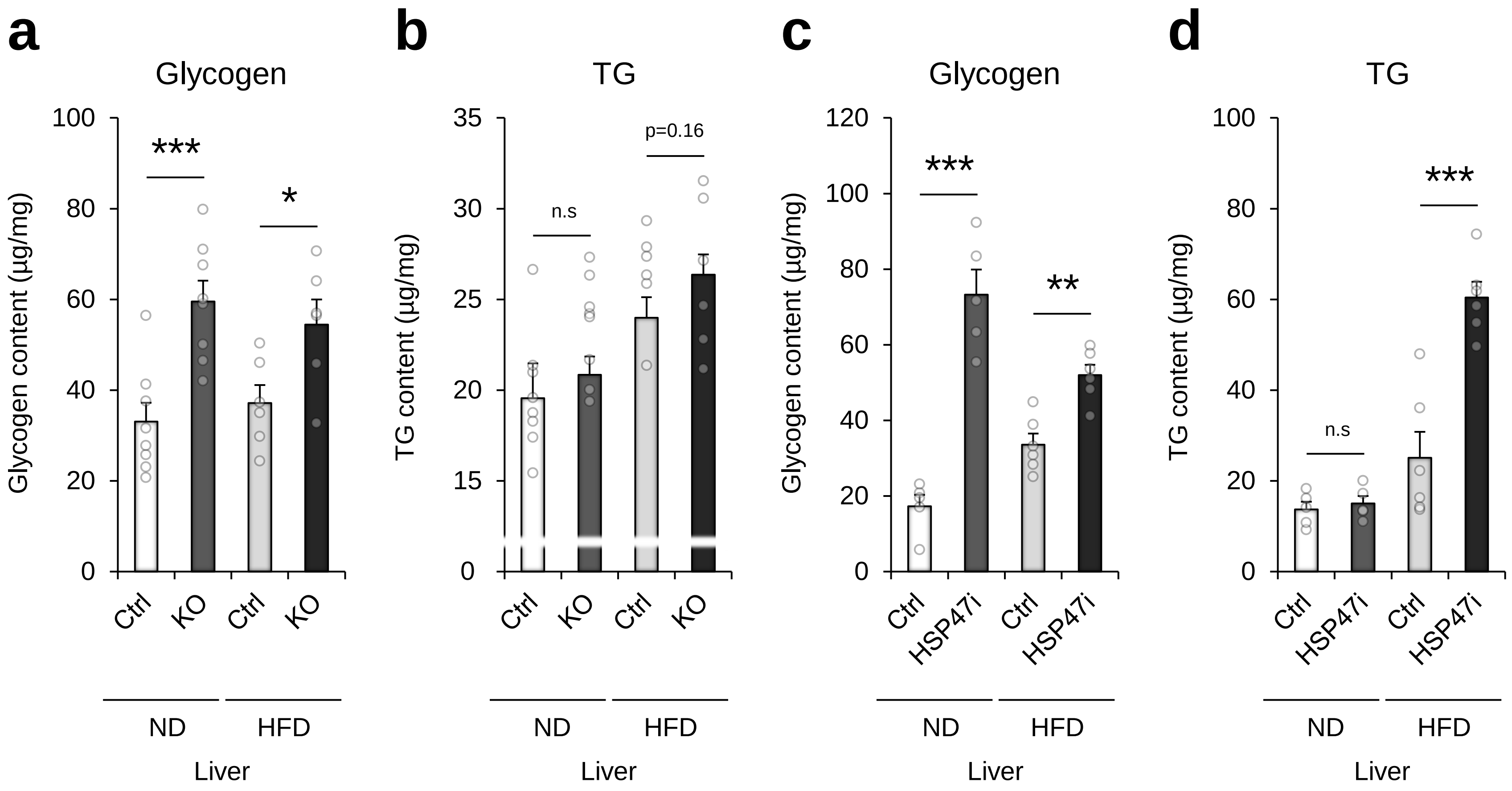

Supplementary Figure 7. Increased glycogen and triglyceride contents in liver of AdHSP47KO and HSP47i mice. a and b, Measurement of glycogen (a; ND, p=0.00075; HFD, p=0.029) and triglyceride (b) contents in liver of AdHSP47KO mice under normal (n=8 each) or 7 days of high-fat diet (n=6 each) condition. c and d, Measurement of glycogen (c; ND, p=5.96e-5; HFD, p=0.001061) and triglyceride (d; HFD, p=0.000349) contents in liver of HSP47i mice (24 hours of fasting followed by 24 hours of normal diet, n= 5 each, or 3 days of high-fat diet refeeding, n=6 each, with or without HSP47 inhibitor). Data represent the mean ± SEM. *p < 0.05, **p < 0.01, and ***p < 0.001; n refers to sample size. ND, normal diet; HFD, high-fat diet. Statistical significance was determined by two-tailed unpaired t-test. Source data are provided as a Source Data file.

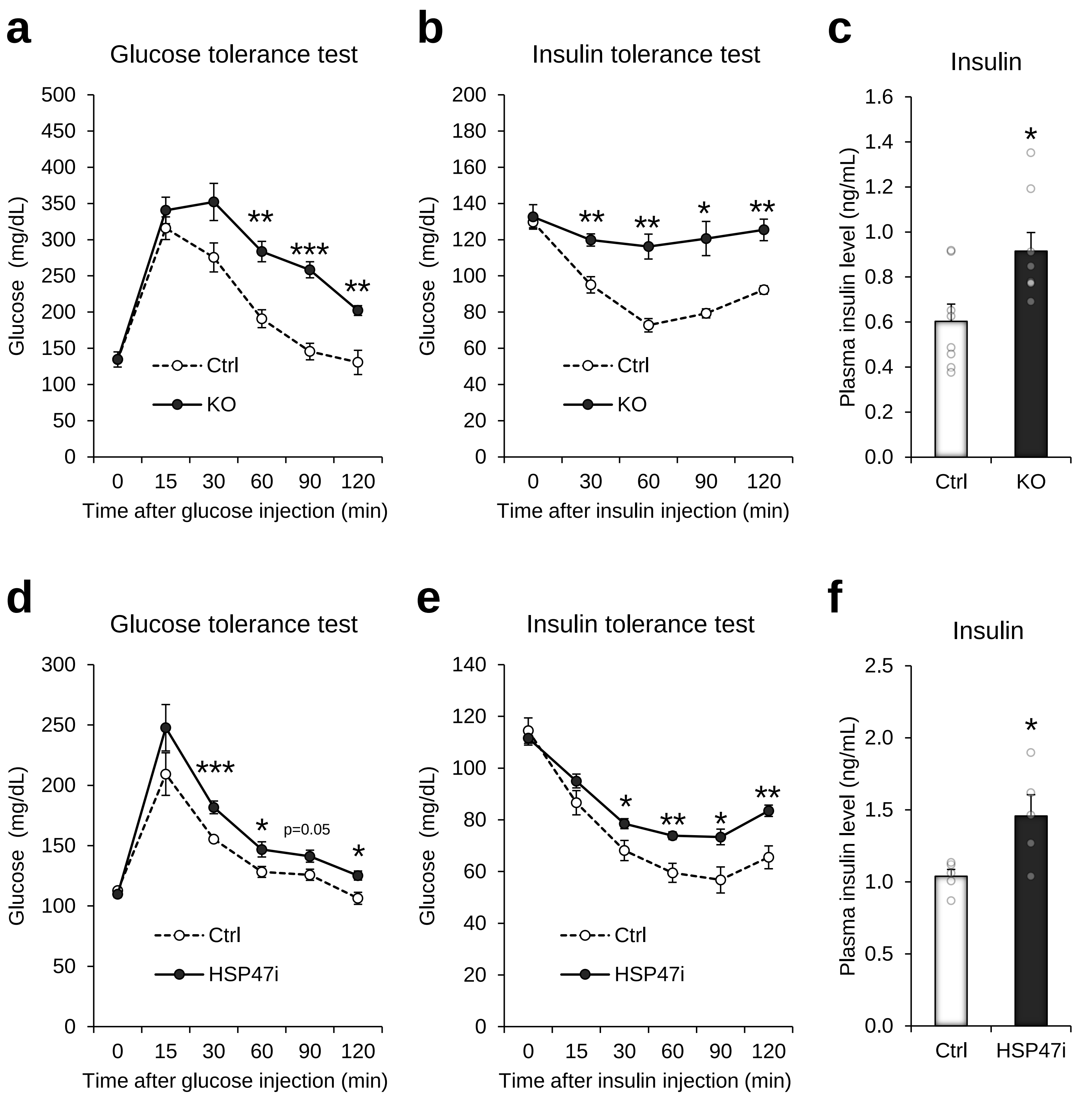

Supplementary Figure 8. Impairment of glucose and insulin tolerance in AdHSP47KO and HSP47i mice. a and b, Glucose (a; 60 min, p=0.003; 90 min, p=0.00032; 120 min, p=0.00398) and insulin (b; 30 min, p=0.0037; 60 min, p=0.00232; 90 min, p=0.0113; 120 min, p=0.00389) tolerance test in control and AdHSP47KO mice (control, Ctrl, n=4; AdHSP47KO, KO, n=5). c, Plasma insulin level in control and AdHSP47KO fasted after 5 hours fasting (n=8 each; p=0.0149). d and e, Glucose (d; 30 min, p=0.000731; 60 min, p=0.041; 120 min, p=0.0133) and insulin (e; 30 min, p=0.0418; 60 min, p=0.00431; 90 min, p=0.0191; 120 min, p=0.00443) tolerance test in control and HSP47i mice (n=8 each). f, Plasma insulin level in control and HSP47i mice fasted after 5 hours fasting (n=5 each; p=0.0264). Data represent the mean ± SEM. *p < 0.05, **p < 0.01, and ***p < 0.001; n refers to sample size. Statistical significance was determined by two-tailed unpaired t-test. Source data are provided as a Source Data file.

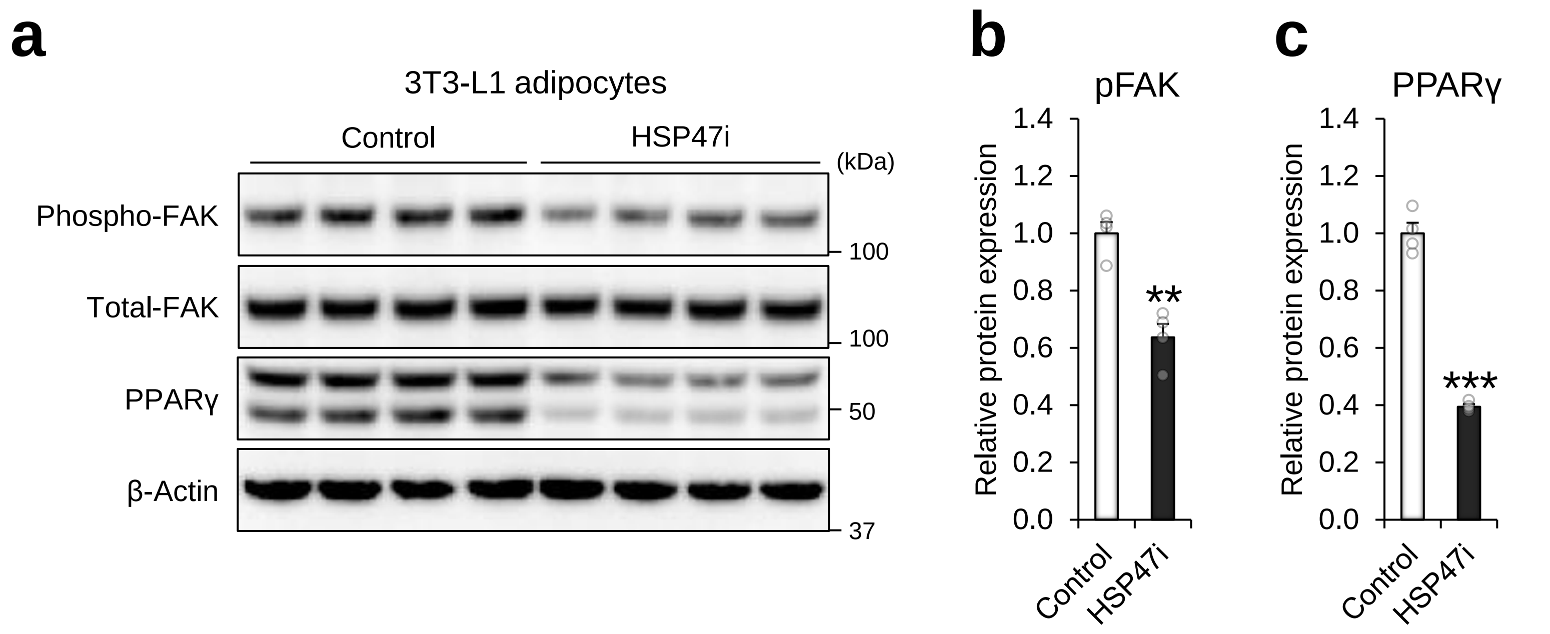

Supplementary Figure 9. HSP47 inhibition in 3T3-L1 adipocytes. a-c, Western blot image (a) of HSP47, FAK signal and PPARγ proteins in 3T3-L1 adipocytes after control or HSP47i (200 μM) for 3 hours; the densitometry of pFAK (b; p=0.001041), and PPARγ (c; p=3.33e-6) proteins. Data represent the mean ± SEM. **$p < 0.01$; n refers to sample size. Statistical significance was determined by two-tailed unpaired t-test. Source data are provided as a Source Data file.

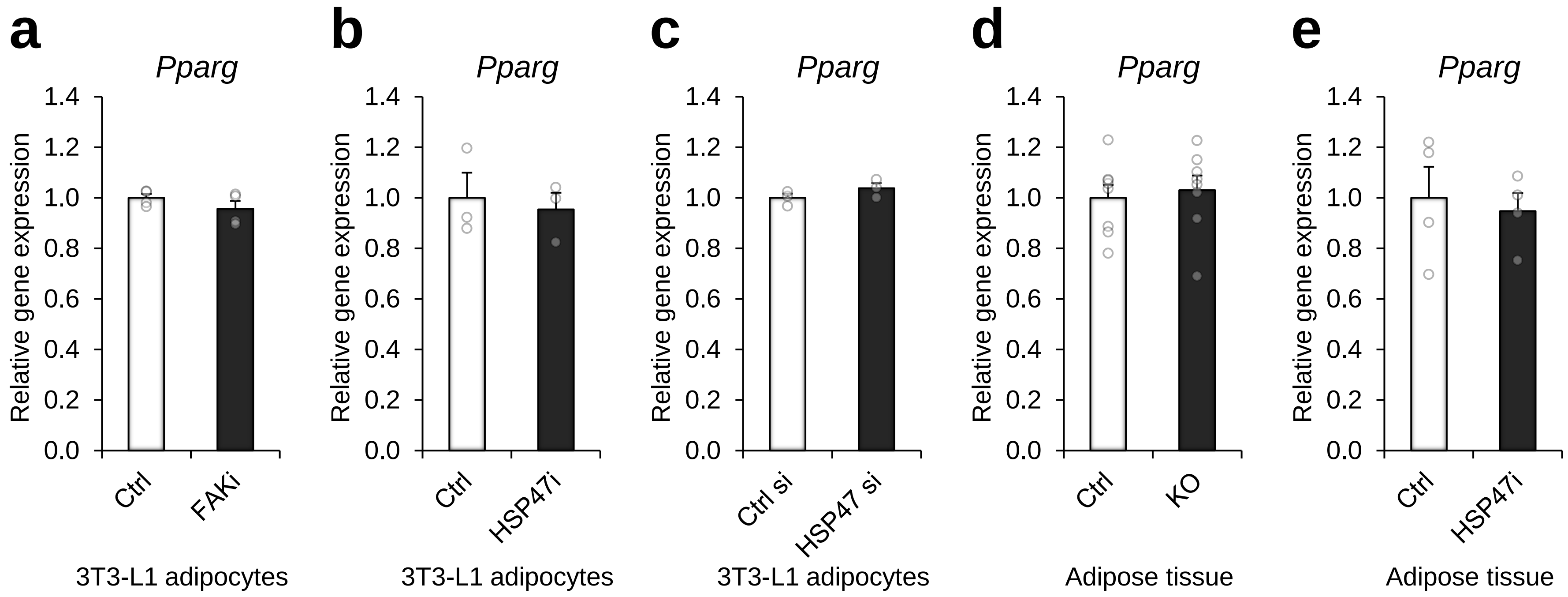

Supplementary Figure 10. HSP47 ablation do not change Pparg gene expression in adipocytes and adipose tissue. a, Relative *Pparg* gene expression in 3T3-L1 adipocytes after FAKi (1 μM) treatment for 3 hours (n=4 each). b, Relative *Pparg* gene expression in 3T3-L1 adipocytes after HSP47i (200 μM) treatment for 3 hours (n=3 each). c, Relative *Pparg* gene expression in 3T3-L1 adipocytes after *Hsp47* siRNA (n=3 each). d, Relative *Pparg* gene expression in mouse adipose tissue of control (Ctrl; n=17) and AdHSP47KO (KO; n=12). e, Relative *Pparg* gene expression in mouse adipose tissue of control (Ctrl; n=4) and HSP47i (KO; n=4). Data represent the mean ± SEM; n refers to sample size. Source data are provided as a Source Data file.

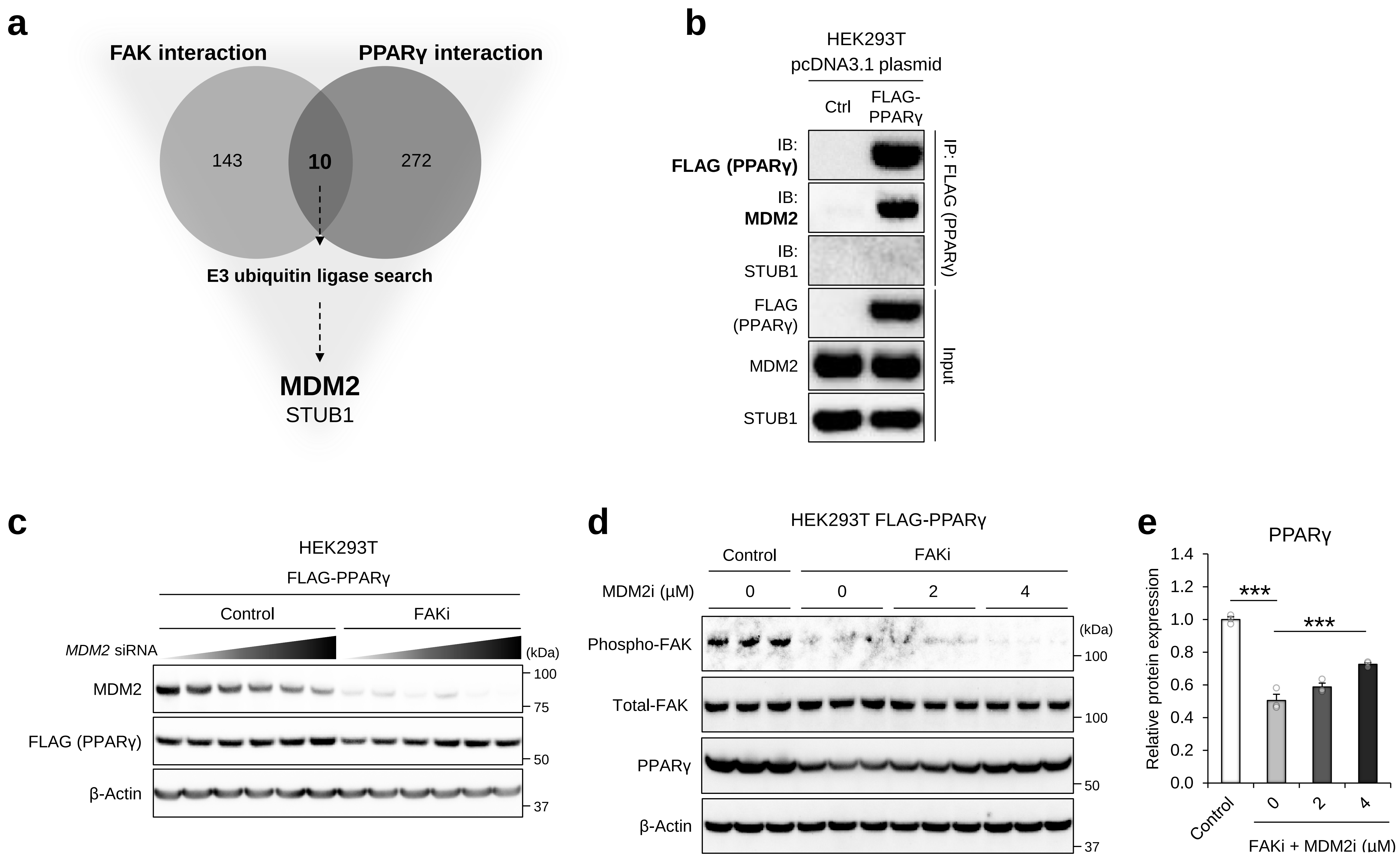

Supplementary Figure 11. MDM2 mediates FAK-linked PPARγ protein control. a, Schematic diagram of screening for E3 ubiquitin ligase that mediates FAK-linked PPARg protein stability control; FAK-interacting proteins (153 proteins) and PPARγ-interacting proteins (282 proteins) were identified in BioGRID, a biomedical interaction repository. b, Western blot image of FLAG-PPARγ, MDM2, and STUB1 proteins in co-immunoprecipitation assay of FLAG-PPARγ-overexpressed HEK293T cells. c, Western blot image of MDM2 and FLAG-PPARγ proteins in FLAG-PPARγ-overexpressed HEK293T cells after 48 hours of control and/or *MDM2* siRNA (0, 2.5, 5, 10, 20, 40 nM; total 40 nM/sample) followed by 3 hours of control or FAKi (10 μM) treatment. d and e, Western blot image (d) of FLAG-PPARγ protein in FLAG-PPARγ-overexpressed HEK293T after FAKi (10 μM) treatment with/without MDM2i (MI-773; 0, 2, and 4 μM) for 3 hours; the densitometry (e; Control vs FAKi, $p<0.0001$; FAKi vs FAKi + MDM2i 4 μM, $p=0.0009$) of PPARγ protein (n=3 each). Data represent the mean ± SEM. ***$p < 0.001$; n refers to sample size. Statistical significance was determined by Tukey-Kramer test. Source data are provided as a Source Data file.

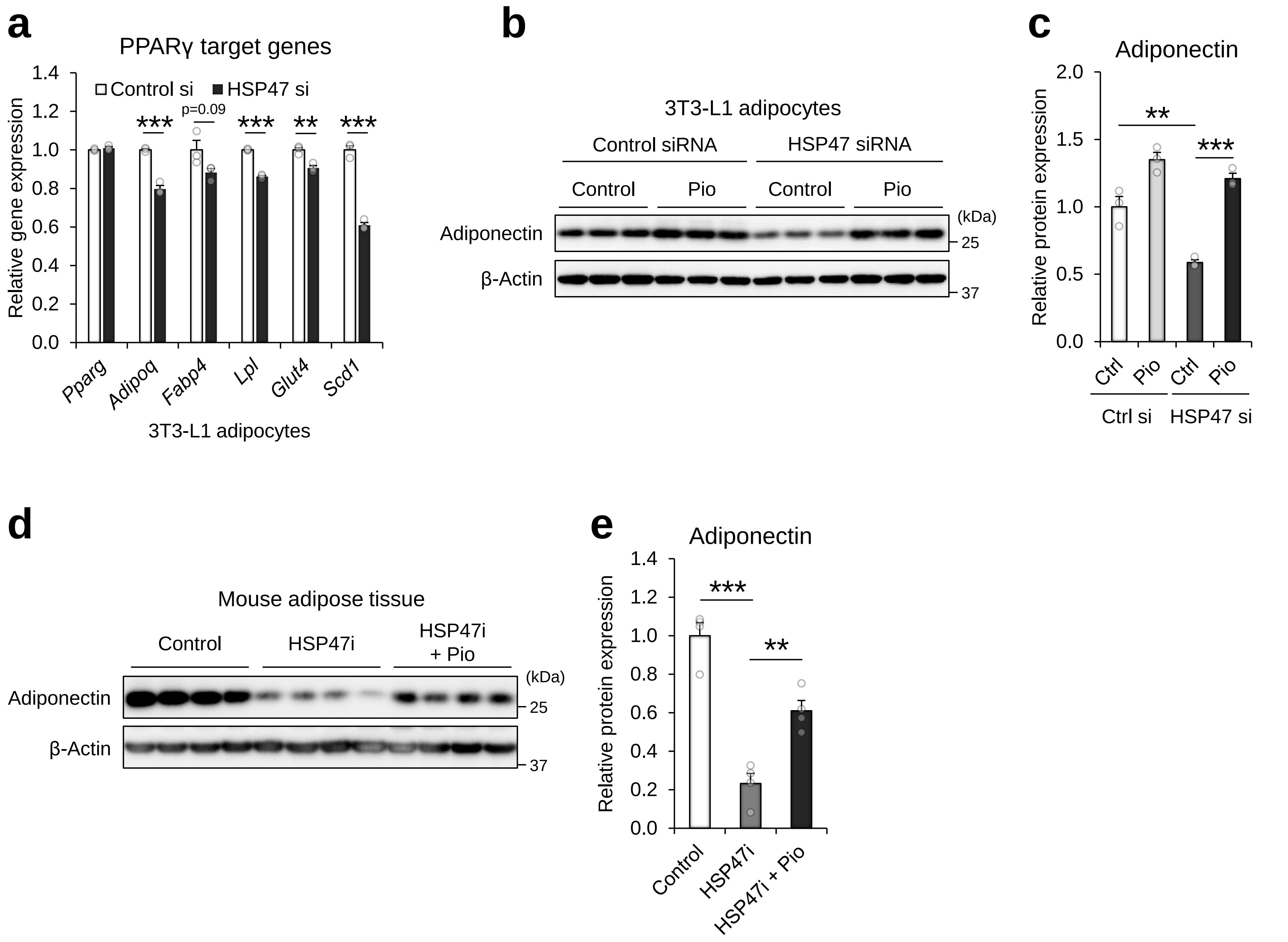

Supplementary Figure 12. HSP47 ablations decrease the activity of PPARγ and adiponectin restoration by pioglitazone treatment. a, Realtime qPCR data of PPARγ and the target genes (*Adipoq*, p=0.00042, *Lpl*, p=2.5e-5, *Glut4*, p=0.000622, *Scd1*, p=0.00012) in 3T3-L1 adipocytes after control or HSP47 siRNA (n=3 each). b and c, Western blot image (b) of adiponectin in 3T3-L1 adipocytes after control or HSP47 siRNA with/without pioglitazone (1 μM; Pio) (n=3 each); the densitometry (c; Ctrl si vs HSP47 si, p=0.0021; HSP47si vs HSP47si + Pio, p=0.0001) of adiponectin protein. d and e, Western blot image (d) of adiponectin in mouse adipose tissue (epididymal) after HSP47i treatment with/without pioglitazone (30 mg/kg; Pio) under fasting/feeding cycle (n=4 each); the densitometry (e; Control vs HSP47i, p<0.0001; HSP47i vs HSP47i + Pio, p=0.0036) of adiponectin protein. Data represent the mean ± SEM. **p < 0.01, and ***p < 0.001; n refers to sample size. Statistical significance was determined by two-tailed unpaired t-test (a) and Tukey-Kramer test (c and e). Source data are provided as a Source Data file.

Supplementary Table 1. Material list

REAGENT or RESOURCE	SOURCE	IDENTIFIER
Animals and cells		
C57BL/6J mice	Charles River Japan	N.A
Nr3c1 (*Gr*; glucocorticoid receptor) floxed mice	Jackson Laboratory	021021
Serpinh1 (*Hsp47*) floxed mice	RIKEN BRC	RBRC10972
Adiponectin-Cre mice	E. Rosen (BIDMC)	N.A
HEK293T cells	ATCC	CRL-3216
3T3-L1 cells	ATCC	CL-173
Deposited data		
Human tissues gene expression[1]	GTEx	N.A
Human tissue and cell protein expression[2]	Proteomics DB	N.A
Human gene variants[3]	GWAS catalog	N.A
Human adipose tissue in fasting and feeding[4]	GEO datasets	GSE154612
Human adipose tissue in overeating (high fat diet)[5]	GEO datasets	GSE28005
Human obese adipocytes[6]	GEO datasets	GDS3602
Human adipose tissue discordant for body fatness[7]	GEO datasets	GSE92405
Human adipose tissue in exercise training[8]	GEO datasets	GSE116801
Human adipose tissue in low-calorie diet[9]	GEO datasets	GSE77962
Human adipose tissue before/after bariatric surgery[10]	GEO datasets	GSE72158
Human adipose tissue with/without cachexia[11]	GEO datasets	GSE20571
Human adipose tissue in METSIM study[12]	GEO datasets	GSE70353
Mouse tissue gene expression[13]	GEO datasets	GDS3142
Mouse adipose tissue in fasting and feeding[14]	GEO datasets	GDS4918
Mouse adipose tissue in overeating (3 days HFD)[15]	GEO datasets	GDS5824
Mouse adipose tissue in 3 months of HFD[16]	GEO datasets	GDS4811
Mouse adipose tissue of high- or low-weight gainer[17]	GEO datasets	GDS2319
Mouse adipose tissue in calorie restriction[18]	GEO datasets	GSE60596
Mouse adipose tissue in exercise training[19]	GEO datasets	GSE70857
Mouse adipose tissue in cancer cachexia[20]	GEO datasets	GDS4899
Rat adipose tissue in fasting and feeding[21]	GEO datasets	GDS3135
Rat adipose tissue in high-fat/high-sucrose diet[22]	GEO datasets	GDS2946
Rat adipose tissue of WNIN/Ob lean and obese[23]	GEO datasets	GSE58575
Rat adipose tissue in starvation[24]	GEO datasets	GSE118978
Rat adipose tissue in mild calorie restriction[25]	GEO datasets	GSE176295
Rat adipose tissue after bariatric surgery (RYGB)[26]	GEO datasets	GDS2956
Chicken adipose tissue in insulin neutralization[27]	GEO datasets	GSE35581
Human adipose explant in glucocorticoid treatment[28]	GEO datasets	GSE88966
Human fibroblast and adipose for eQTL analysis[1]	GTEx	N.A
FAK and PPAR interacting proteins[29]	BioGrid	N.A
SNPs allele frequencies[30]	1000 genome project	N.A
Antibody		
Anti-PPARγ (81B8)	Cell Signaling Technology	#2443
Anti-Phospho-FAK (Tyr397) (D20B1)	Cell Signaling Technology	#8556S

Anti-Total FAK	Cell Signaling Technology	#3285
Anti-Total FAK (clone 4.47)	Merck Millipore	05-537
Anti-Collagen VI	Abcam	ab182744
Anti-α-Tubulin (11H10)	Cell Signaling Technology	#2125
Anti-β-Actin	Sigma-Aldrich	A5441
Anti-MDM2 (D1V2Z)	Cell Signaling Technology	#86934
Anti-Adiponectin	R&D system	MAB3100
Anti-FLAG (M2)	Sigma-Aldrich	F1804
Anti-FLAG (M2)-HRP	Sigma-Aldrich	A8592
Anti-HA-Tag (C29F4)	Cell Signaling Technology	#3724
Anti-Rabbit IgG, Horseradish Peroxidase	Amersham	NA934V
Anti-Mouse IgG, Horseradish Peroxidase	Amersham	NA931V
Anti-Rat IgG, Horseradish Peroxidase	Amersham	NA935V
Goat anti-Rabbit IgG (H+L)-Alexa Fluor 555	Invitrogen	A-11008
Plasmid		
pRK5-HA-Ubiquitin-WT	Addgene	17608
pcDNA3.1-PPARγ	Addgene	8895
Reagent		
Col003 (HSP47 inhibitor; HSP47i)	Sigma-Aldrich	SML2264
PF-573228 (FAK inhibitor; FAKi)	Selleck	S2013
RGD peptide	Santa Cruz	114681-65-1
MI-773 (MDM2 inhibitor; MDM2i)	Selleck	S7649
MG132 (Proteasome inhibitor)	Sigma-Aldrich	M7449
OSI-906 (Insulin receptor inhibitor; IRi)	Selleck	S1091
RU486 (Glucocorticoid receptor inhibitor; GRi)	Selleck	S2606
TRI Reagent	Sigma-Aldrich	T9424
Transcriptor Universal cDNA Master	Roche	05893151001
FastStart Essential DNA Green Master	Roche	06924204001
Lipofectamine 3000	Thermo Fisher Scientific	L3000015
Lipofectamine™ RNAiMAX	Thermo Fisher Scientific	13778100
PVDF (0.2 nm)	BIO-RAD	#1620177
RIPA buffer	Nacalai tesque	16488-34
Protease inhibitor cocktail	Nacalai tesque	25955
Phosphatase inhibitor cocktail	Nacalai tesque	07575-51
4% PFA	Nacalai tesque	09154-85
Pioglitazone	Sigma-Aldrich	CDS021593
Allstar Negative control (Control siRNA)	Qiagen	1027280
Silencer™ Select Negative Control (Control siRNA)	Invitrogen	4390843
Hsp47 siRNA	Qiagen	SI01415134
Itgb1 siRNA	Qiagen	SI02670437
MDM2 siRNA	Invitrogen	4390824
BODIPY™ 493/503	Invitrogen	D3922
DAPI	AAT Bioquest	17507
Fluoromount-G™ Mounting Medium	Invitrogen	00-4958-02
Cell Imaging Plates, size 96 wells, glass bottom	Eppendorf	0030741030

DTSSP	Thermo Fisher Scientific	21578
ANTI-FLAG® M2 Affinity Gel	Sigma-Aldrich	A2220
Dynabeads™ Protein G	Thermo Fisher Scientific	10003D
FLAG peptide	Sigma-Aldrich	F3290
Pierce Western Blotting Substrate Plus	Thermo Fisher Scientific	32132
Pierce™ BCA Protein Assay Kit	Thermo Fisher Scientific	23225
Mouse Insulin ELISA Kit	Morinaga	MS303
Triglyceride E-test	Wako	432-40201
Glycogen Assay Kit	Cayman	700480
High-fat diet (60 kcal%)	Research Diets	D12492
SDS sample buffer solution	Fujifilm Wako	198-13282
RT-qPCR primer		
Rplp0 Fw: GCTCCAAGCAGATGCAGCA	This paper	N.A
Rplp0 Rv: CCGGATGTGAGGCAGCAG	This paper	N.A
Cyclophilin Fw: CAGACGCCACTGTCGCTTT	This paper	N.A
Cyclophilin Rv: TGTCTTTGGAACTTTGTCTGCAA	This paper	N.A
Pparg Fw: ATCTTAACTGCCGGATCCACAA	This paper	N.A
Pparg Rv: GCCCAAACCTGATGGCATT	This paper	N.A
Adipoq Fw: GATGGCAGAGATGGCACTCC	This paper	N.A
Adipoq Rv: CTTGCCAGTGCTGCCGTCAT	This paper	N.A
Fabp4 Fw: CCGCAGACGACAGGA	This paper	N.A
Fabp4 Rv: CTCATGCCCTTTCATAAACT	This paper	N.A
Cd36 Fw; GATGTGGAACCCATAACTGGATTCAC	This paper	N.A
Cd36 Rv: GGTCCCAGTCTCATTTAGCCACAGTA	This paper	N.A
Lpl Fw: CCCTGAAGACACAGCTGAGG	This paper	N.A
Lpl Rv: GGCTGTACCCTAAGAGGTGG	This paper	N.A
Glut4 Fw: GCGGATGCTATGGGTCCTTA	This paper	N.A
Glut4 Rv: GTCCGGCCTCTGGTTTCAG	This paper	N.A
Scd1 Fw: TGGGTTGGCTGCTTGTG	This paper	N.A
Scd1 Rv: GCGTGGGCAGGATGAAG	This paper	N.A
Tools and Software		
LightCycler® 96 System and software	Roche	05815916001
ChemiDoc Touch imaging system	BIO-RAD	17001402
LSM880	ZEISS	
GEO2R	https://www.ncbi.nlm.nih.gov/geo/geo2r/	N.A
Microsoft Excel	https://www.microsoft.com/	N.A
Toppgene functional analysis software (ToppFun)	https://toppgene.cchmc.org/	N.A
JMP Pro 15.2.1 software	https://www.jmp.com/	N.A
ZEN (2.3 lite)	https://www.zeiss.co.jp	N.A
ImageJ	https://imagej.nih.gov/ij/	N.A

Reference

1 Lonsdale, J. *et al.* The Genotype-Tissue Expression (GTEx) project. *Nature Genetics* **45**, 580-585, doi:10.1038/ng.2653 (2013).

2 Wilhelm, M. *et al.* Mass-spectrometry-based draft of the human proteome. *Nature* **509**, 582-587, doi:10.1038/nature13319 (2014).

3 Welter, D. *et al.* The NHGRI GWAS Catalog, a curated resource of SNP-trait associations. *Nucleic acids research* **42**, D1001-D1006 (2014).

4 Defour, M., Michielsen, C., O'Donovan, S. D., Afman, L. A. & Kersten, S. Transcriptomic signature of fasting in human adipose tissue. *Physiol Genomics* **52**, 451-467, doi:10.1152/physiolgenomics.00083.2020 (2020).

5 Alligier, M. *et al.* Subcutaneous adipose tissue remodeling during the initial phase of weight gain induced by overfeeding in humans. *J Clin Endocrinol Metab* **97**, E183-192, doi:10.1210/jc.2011-2314 (2012).

6 Lee, Y. H. *et al.* Microarray profiling of isolated abdominal subcutaneous adipocytes from obese vs non-obese Pima Indians: increased expression of inflammation-related genes. *Diabetologia* **48**, 1776-1783, doi:10.1007/s00125-005-1867-3 (2005).

7 Heinonen, S. *et al.* Mitochondria-related transcriptional signature is downregulated in adipocytes in obesity: a study of young healthy MZ twins. *Diabetologia* **60**, 169-181, doi:10.1007/s00125-016-4121-2 (2017).

8 Takahashi, H. *et al.* TGF-β2 is an exercise-induced adipokine that regulates glucose and fatty acid metabolism. *Nature metabolism* **1**, 291-303 (2019).

9 Vink, R. G. *et al.* Adipose tissue gene expression is differentially regulated with different rates of weight loss in overweight and obese humans. *Int J Obes (Lond)* **41**, 309-316, doi:10.1038/ijo.2016.201 (2017).

10 Liu, Y. *et al.* Accumulation and Changes in Composition of Collagens in Subcutaneous Adipose Tissue After Bariatric Surgery. *The Journal of Clinical Endocrinology & Metabolism* **101**, 293-304, doi:10.1210/jc.2015-3348 (2016).

11 Dahlman, I. *et al.* Adipose tissue pathways involved in weight loss of cancer cachexia. *Br J Cancer* **102**, 1541-1548, doi:10.1038/sj.bjc.6605665 (2010).

12 Civelek, M. *et al.* Genetic Regulation of Adipose Gene Expression and Cardio-Metabolic Traits. *Am J Hum Genet* **100**, 428-443, doi:10.1016/j.ajhg.2017.01.027 (2017).

13 Thorrez, L. *et al.* Using ribosomal protein genes as reference: a tale of caution. *PLoS One* **3**, e1854, doi:10.1371/journal.pone.0001854 (2008).

14 Schupp, M. *et al.* Metabolite and transcriptome analysis during fasting suggest a role for the p53-Ddit4 axis in major metabolic tissues. *BMC Genomics* **14**, 758, doi:10.1186/1471-2164-14-758 (2013).

15 Kim, J. I. *et al.* Lipid-overloaded enlarged adipocytes provoke insulin resistance independent of inflammation. *Mol Cell Biol* **35**, 1686-1699, doi:10.1128/mcb.01321-14 (2015).

16 Ichimura, A. *et al.* Dysfunction of lipid sensor GPR120 leads to obesity in both mouse and human. *Nature* **483**, 350-354, doi:10.1038/nature10798 (2012).

17 Koza, R. A. *et al.* Changes in gene expression foreshadow diet-induced obesity in genetically identical mice. *PLoS Genet* **2**, e81, doi:10.1371/journal.pgen.0020081 (2006).

18 Kim, S. S. *et al.* Whole-transcriptome analysis of mouse adipose tissue in response to short-term caloric restriction. *Mol Genet Genomics* **291**, 831-847, doi:10.1007/s00438-015-1150-3 (2016).

19 Boutant, M. *et al.* SIRT1 gain of function does not mimic or enhance the adaptations to intermittent fasting. *Cell reports* **14**, 2068-2075 (2016).

20 Gilabert, M. *et al.* Pancreatic cancer-induced cachexia is Jak2-dependent in mice. *J Cell Physiol* **229**, 1437-1443, doi:10.1002/jcp.24580 (2014).

21 Nakai, Y. *et al.* Up-regulation of genes related to the ubiquitin-proteasome system in the brown adipose tissue of 24-h-fasted rats. *Biosci Biotechnol Biochem* **72**, 139-148, doi:10.1271/bbb.70508 (2008).

22 Li, S. *et al.* Assessment of diet-induced obese rats as an obesity model by comparative functional genomics. *Obesity (Silver Spring)* **16**, 811-818, doi:10.1038/oby.2007.116 (2008).

23 Sakamuri, S. S. V. P., Putcha, U. K., Veettil, G. N. & Ayyalasomayajula, V. Transcriptome profiling of visceral adipose tissue in a novel obese rat model, WNIN/Ob & its comparison with other animal models. *The Indian Journal of Medical Research* **144**, 409 (2016).

24 Ibrahim, M. *et al.* Alterations in rat adipose tissue transcriptome and proteome in response to prolonged fasting. *Biol Chem* **401**, 389-405, doi:10.1515/hsz-2019-0184 (2020).

25 Saito, K., Ito, M., Chiba, T., Jia, H. & Kato, H. A Comparison of Gene Expression Profiles of Rat Tissues after Mild and Short-Term Calorie Restrictions. *Nutrients* **13**, doi:10.3390/nu13072277 (2021).

26 Guijarro, A. *et al.* Characterization of weight loss and weight regain mechanisms after Roux-en-Y gastric bypass in rats. *Am J Physiol Regul Integr Comp Physiol* **293**, R1474-1489, doi:10.1152/ajpregu.00171.2007 (2007).

27 Ji, B. *et al.* Transcriptomic and metabolomic profiling of chicken adipose tissue in response to insulin neutralization and fasting. *BMC Genomics* **13**, 441, doi:10.1186/1471-2164-13-441 (2012).

28 Pickering, R. T., Lee, M. J., Karastergiou, K., Gower, A. & Fried, S. K. Depot Dependent Effects of Dexamethasone on Gene Expression in Human Omental and Abdominal Subcutaneous Adipose Tissues from Obese Women. *PLoS One* **11**, e0167337, doi:10.1371/journal.pone.0167337 (2016).

29 Stark, C. *et al.* BioGRID: a general repository for interaction datasets. *Nucleic acids research* **34**, D535-D539 (2006).

30 Siva, N. 1000 Genomes project. *Nature biotechnology* **26**, 256-257 (2008).
