# Supplementary material for: HSP47 levels determine the degree of body adiposity
Source: Nat Commun. 2023 Nov 11;14:7319. doi: 10.1038/s41467-023-43080-x (PMC10640548; doi:10.1038/s41467-023-43080-x)
Supplement: Supplementary file 3 — Reporting Summary [file 41467_2023_43080_MOESM3_ESM.pdf]

nature portfolio

Corresponding author(s): Jihoon Shin

Last updated by author(s): Oct 23, 2023

Reporting Summary

Nature Portfolio wishes to improve the reproducibility of the work that we publish. This form provides structure for consistency and transparency in reporting. For further information on Nature Portfolio policies, see our Editorial Policies and the Editorial Policy Checklist.

Statistics

For all statistical analyses, confirm that the following items are present in the figure legend, table legend, main text, or Methods section.

n/a	Confirmed	
☐	☒	The exact sample size (*n*) for each experimental group/condition, given as a discrete number and unit of measurement
☐	☒	A statement on whether measurements were taken from distinct samples or whether the same sample was measured repeatedly
☐	☒	The statistical test(s) used AND whether they are one- or two-sided<br>*Only common tests should be described solely by name; describe more complex techniques in the Methods section.*
☒	☐	A description of all covariates tested
☒	☐	A description of any assumptions or corrections, such as tests of normality and adjustment for multiple comparisons
☐	☒	A full description of the statistical parameters including central tendency (e.g. means) or other basic estimates (e.g. regression coefficient) AND variation (e.g. standard deviation) or associated estimates of uncertainty (e.g. confidence intervals)
☐	☒	For null hypothesis testing, the test statistic (e.g. *F*, *t*, *r*) with confidence intervals, effect sizes, degrees of freedom and *P* value noted<br>*Give P values as exact values whenever suitable.*
☒	☐	For Bayesian analysis, information on the choice of priors and Markov chain Monte Carlo settings
☒	☐	For hierarchical and complex designs, identification of the appropriate level for tests and full reporting of outcomes
☒	☐	Estimates of effect sizes (e.g. Cohen's *d*, Pearson's *r*), indicating how they were calculated

Our web collection on statistics for biologists contains articles on many of the points above.

Software and code

Policy information about availability of computer code

Data collection	Microsoft Excel (Office 2016).
Data analysis	JMP pro 17 was used for statistical analysis. ImageJ (v1.52a) was used for quantification of Western blots. Zen 3.4 (blue) was used for confocal microscopy image and cell size. Enriched pathways in the datasets were analyzed using STRING (v11.0b and 11.5). Details are in the Methods.

For manuscripts utilizing custom algorithms or software that are central to the research but not yet described in published literature, software must be made available to editors and reviewers. We strongly encourage code deposition in a community repository (e.g. GitHub). See the Nature Portfolio guidelines for submitting code & software for further information.

Data

Policy information about availability of data

All manuscripts must include a data availability statement. This statement should provide the following information, where applicable:

- Accession codes, unique identifiers, or web links for publicly available datasets
- A description of any restrictions on data availability
- For clinical datasets or third party data, please ensure that the statement adheres to our policy

Source data are provided with this paper. GEO, GTEx, and other database used in this paper are listed in supplementary table1.

Field-specific reporting

Please select the one below that is the best fit for your research. If you are not sure, read the appropriate sections before making your selection.

- [x] Life sciences
- [ ] Behavioural & social sciences
- [ ] Ecological, evolutionary & environmental sciences

For a reference copy of the document with all sections, see nature.com/documents/nr-reporting-summary-flat.pdf

Life sciences study design

All studies must disclose on these points even when the disclosure is negative.

Sample size	The sample size used in a study is usually determined based on the need to offer sufficient statistical power. The sample size for each experiment is detailed in the figure legends.
Data exclusions	In the transcriptomic analyses, we removed poor quality samples before the analysis, but this involved only 1 or 2 samples of the entire data set. Except for this, no data were excluded from the analyses.
Replication	All measures were replicated at least 2 times and in most cases 3 or more. N values for replicates are reported throughout the manuscript.
Randomization	All cells were randomly assigned to experimental group.
Blinding	The use of different cell lines (3T3-L1, HEK293T) and different treatments at various concentration necessitated the studies to be unblind. However, everything was made to perform each measure and analysis in an unbiased manner.

Reporting for specific materials, systems and methods

We require information from authors about some types of materials, experimental systems and methods used in many studies. Here, indicate whether each material, system or method listed is relevant to your study. If you are not sure if a list item applies to your research, read the appropriate section before selecting a response.

Materials & experimental systems

n/a	Involved in the study	
[ ]	[x]	Antibodies
[ ]	[x]	Eukaryotic cell lines
[x]	[ ]	Palaeontology and archaeology
[ ]	[x]	Animals and other organisms
[x]	[ ]	Human research participants
[x]	[ ]	Clinical data
[x]	[ ]	Dual use research of concern

Methods

n/a	Involved in the study	
[x]	[ ]	ChIP-seq
[x]	[ ]	Flow cytometry
[x]	[ ]	MRI-based neuroimaging

Antibodies

Antibodies used

The following antibodies were used in this study:
Anti-PPARγ (81B8)(Cell Signaling Technology #2443; 1:1000 dilution)
Anti-Phospho-FAK (Tyr397) (D20B1)(Cell Signaling Technology #8556S; 1:1000 dilution)
Anti-Total FAK (Cell Signaling Technology #3285; 1:1000 dilution)
Anti-Total FAK (clone 4.47)(Merck Millipore 05-537; 1:1000 dilution)
Anti-Collagen VI (Abcam ab182744; 1:1000 dilution)
Anti-α-Tubulin (11H10) (Cell Signaling Technology #2125; 1:1000 dilution)
Anti-β-Actin (Sigma-Aldrich A5441; 1:5000 dilution)
Anti-MDM2 (D1V2Z) (Cell Signaling Technology #86934; 1:1000 dilution)
Anti-Adiponectin (R&D system MAB3100; 1:4000 dilution)
Anti-FLAG (M2) (Sigma-Aldrich F1804; 1:3000 dilution)
Anti-FLAG (M2)-HRP (Sigma-Aldrich A8592; 1:1000 dilution)
Anti-HA-Tag (C29F4) (Cell Signaling Technology #3724; 1:1000 dilution)
Anti-Rabbit IgG, Horseradish Peroxidase (Amersham NA934V; 1:1000 dilution)
Anti-Mouse IgG, Horseradish Peroxidase (Amersham NA931V; 1:1000 dilution)
Anti-Rat IgG, Horseradish Peroxidase (Amersham NA935V; 1:1000 dilution)
Goat anti-Rabbit IgG (H+L)-Alexa Fluor 555 (Invitrogen A-11008; 1:1000 dilution)

Validation

All antibodies used in this study were comercially developed and used in previous studies. Information regarding validation and application can be found on manufacturer's website as follows.

Anti-PPARγ (https://www.cellsignal.com/products/primary-antibodies/pparg-81b8-rabbit-mab/2443)
Anti-Phospho-FAK (https://www.cellsignal.com/products/primary-antibodies/phospho-fak-tyr397-d20b1-rabbit-mab/8556)
Anti-Total FAK (https://www.cellsignal.com/products/primary-antibodies/fak-antibody/3285)
Anti-Collagen VI (https://www.abcam.com/products/primary-antibodies/collagen-vi-antibody-epr17072-ab182744.html)
Anti-α-Tubulin (https://www.cellsignal.com/products/primary-antibodies/a-tubulin-11h10-rabbit-mab/2125)
Anti-β-Actin (https://www.sigmaaldrich.com/US/en/product/sigma/a5441)
Anti-MDM2 (https://www.cellsignal.com/products/primary-antibodies/mdm2-d1v2z-rabbit-mab/86934)
Anti-Adiponectin (https://www.rndsystems.com/products/rat-adiponectin-acrp30-antibody-861812_mab3100)
Anti-FLAG (https://www.sigmaaldrich.com/US/en/product/sigma/f1804)
Anti-FLAG (M2)-HRP (https://www.sigmaaldrich.com/US/en/product/sigma/a8592)
Anti-HA-Tag (https://www.cellsignal.com/products/primary-antibodies/ha-tag-c29f4-rabbit-mab/3724)
Anti-Rabbit IgG, Horseradish Peroxidase (https://www.cytivalifesciences.com/en/us/shop/protein-analysis/blotting-and-detection/blotting-standards-and-reagents/amersham-ecl-hrp-conjugated-antibodies-p-06260)
Anti-Mouse IgG, Horseradish Peroxidase (https://www.cytivalifesciences.com/en/us/shop/protein-analysis/blotting-and-detection/blotting-standards-and-reagents/amersham-ecl-hrp-conjugated-antibodies-p-06260)
Anti-Rat IgG, Horseradish Peroxidase (https://www.cytivalifesciences.com/en/us/shop/protein-analysis/blotting-and-detection/blotting-standards-and-reagents/amersham-ecl-hrp-conjugated-antibodies-p-06260)
Goat anti-Rabbit IgG (H+L)-Alexa Fluor 555 (https://www.thermofisher.com/antibody/product/Goat-anti-Rabbit-IgG-H-L-Cross-Adsorbed-Secondary-Antibody-Polyclonal/A-21428)

Eukaryotic cell lines

Policy information about cell lines

Cell line source(s)	3T3-L1 (CL-173) and 293T (CRL-3216) cells were purchased from ATCC.
Authentication	N/A
Mycoplasma contamination	The cell lines were tested negatively for mycoplasma.
Commonly misidentified lines (See ICLAC register)	N/A

Animals and other organisms

Policy information about studies involving animals; ARRIVE guidelines recommended for reporting animal research

Laboratory animals	C57BL/6J mice were purchased from Charles River Japan (Yokohama, Japan). Adiponectin-Cre mice were provided by E. Rosen (Beth Israel Deaconess Medical Center). Nr3c1 (Gr; glucocorticoid receptor) floxed mice were purchased from the Jackson Laboratory (stock no. 021021). Serpinh1 (Hsp47) floxed mice were purchased from RIKEN BioResource Research Center (RIKEN BRC; BRC No. RBRC10972). Both male and female mice (2-7 months old) were utilized for corresponding analyses.
Wild animals	The study did not involve wild animals.
Field-collected samples	the study did not involve samples collected from the field.
Ethics oversight	All procedures were approved by Osaka University Institutional Animal Care and Use Committee and fully complied with all national and local policies.

Note that full information on the approval of the study protocol must also be provided in the manuscript.
